# Immune Tuning in Extreme Environments: Protein Citrullinome and Extracellular Vesicle Signatures Comparing Hibernating Versus Active States in the Heterothermic and Heterometabolic Tenrec (*Tenrec ecaudatus*)

**DOI:** 10.3390/biology14081056

**Published:** 2025-08-15

**Authors:** Gilbecca Rae Smith, Pinar Uysal-Onganer, Igor Kraev, Frank van Breukelen, Sigrun Lange

**Affiliations:** 1 School of Life Sciences, University of Nevada, Las Vegas, Las Vegas, NV 89154, USA; gilbeccarae.smith@unlv.edu (G.R.S.); frank.vanbreukelen@unlv.edu (F.v.B.); 2 Cancer Mechanisms and Biomarkers Research Group, School of Life Sciences, College of Liberal Arts and Sciences, University of Westminster, 115 New Cavendish Street, London W1W 6UW, UK; p.onganer@westminster.ac.uk; 3 Electron Microscopy Suite, Faculty of Science, Technology, Engineering and Mathematics, Open University, Milton Keynes MK7 6AA, UK; igor.kraev@open.ac.uk; 4 Pathobiology and Extracellular Vesicles Research Group, School of Life Sciences, College of Liberal Arts and Sciences, University of Westminster, 115 New Cavendish Street, London W1W 6UW, UK

**Keywords:** tenrec, extracellular vesicles (EVs), peptidylarginine deiminase (PAD), citrullination/deimination, histone H3 citrullination (CitH3), NETosis, inflammasome, microRNAs, hibernation, extreme environments

## Abstract

Tenrecs are heterothermic burrowing mammals, which are capable of withstanding extreme environmental stressors, including during hibernation. Their phylogenetic position as reminiscent of an ancestral placental mammal makes tenrecs a unique model for evolutionarily conserved traits, with potential translatability to human physiology and pathobiology, including adaptations to extreme environments. In this study, we compared tenrec plasma for post-translational protein citrullination profiles (citrullinomes) and extracellular vesicle (EV) characteristics, including selected microRNA cargoes (miR-21, miR-155, miR-206, miR-210), between baseline active and hibernating states at low (12 °C) and high (28 °C) ambient temperatures. Our findings show considerable changes in citrullinome plasma profiles and associated Gene Ontology and KEGG pathway analysis linked to physiological and inflammatory processes, comparing hibernating and active states, also differing between the two ambient temperature groups. We furthermore identified modified EV profiles with respect to stress-related (miR-21, miR-155), hypoxia (miR-210) and metabolic/muscle related (miR-206) microRNA cargoes, which showed significant differences between active and hibernating animals, also comparing the two ambient temperature groups. Our findings show novel roles for post-translational protein citrullination in regulating immune and metabolic associated pathways in the tenrec, and highlight EV profiles, based on microRNA cargoes, as indicators for stress and metabolic responses in active versus hibernating states, including at different temperatures. Collectively our data highlights the tenrec as an evolutionary model for regulating pathobiological responses in extreme environments and may have translatable potential for human physiology and pathologies.

## Simple Summary

Tenrecs are ancient mammals that hibernate and are used as a model species for survival in extreme environments. Tenrecs have unusual inflammatory, regenerative and metabolic responses, and studying these animals can, therefore, provide valuable insights into human disease mechanisms. This study compared tenrecs at baseline under hibernating and active conditions, at low (12 °C) and high (28 °C) ambient temperatures. We assessed changes in protein citrullination/deimination, which is a post-translational modification that can change protein function and influence inflammatory responses. We also assessed changes in extracellular vesicle (EV) signatures, which are small membrane particles which carry cargoes, including small RNA species, between cells as a key cellular communication mechanism. Our results show that various physiological, cell regulatory, immune and inflammatory pathways are influenced by protein citrullination, with differences between baseline hibernating versus active states and ambient temperatures. There was also a considerable modification of EV cargo content relating to inflammatory, metabolic and stress responses comparing hibernating to active tenrecs, as well as comparing low and high ambient temperatures. Findings in mammals with unusual physiological responses and the ability to hibernate are of considerable translational value for furthering our understanding of human health and survival in extreme environments, including for future human deep space travel.

## 1. Introduction

Tenrecs are heterothermic fossorial mammals, which use burrows during both their active and hibernating seasons in Madagascar and neighbouring islands. Tenrecs are capable of withstanding extreme environmental stressors, including hypoxia and hypercapnia during hibernation [1]. Their phylogenetic position, as reminiscent of an ancestral placental mammal, makes tenrecs a unique model for evolutionarily conserved traits, with possible translatable potential to human physiology and pathobiology, including adaptations to extreme environments [1,2].

From an evolutionary point, the deep rooting of placental mammals is unresolved and significant support exists for at least three very different origins of placental mammals including one scenario where Afrotherians (which includes tenrecs, elephants, manatees and hyraxes) gave rise to other placental mammals (e.g., Exafroplacentalia rooting; [3]). One of the more peculiar Afrotherians is the common tenrec, *Tenrec ecaudatus*. This protoendothermic placental mammal has many features that makes it reminiscent of an ancestral mammal [1,2,4] with traits that may inform physiological and pathobiological processes as even in the active state, body temperature (Tb) may vary by 20 °C and resting oxygen consumption rates may vary by 25-fold [2]. This contrasts with homeostatic regulation seen in modern mammals (Boreoeutherians) where metabolism and body temperature are more consistently stable and high. In spite of being placental mammals, features of the tenrec include a true endodermal cloaca, which is a common urogenital opening normally found in fishes, amphibians, reptiles and birds. Furthermore, tenrecs have the smallest brain of all extant mammals and lack a functional corpus callosum [5]. Other morphological features such as a lack of auditory bullae and zygomatic arches, internal testes just caudal to the kidneys and embryology more reminiscent of a monotreme than a marsupial, complement physiological and life history features such as enormous litter sizes (up to 32 confirmed young in a litter), indeterminate growth, superfetation and hibernation use. Collectively, these features suggest that *Tenrec ecaudatus* may be more plesiomorphic than even *Schrëwdinger*, our hypothetical placental mammal ancestor reconstructed using 4541 morphological characteristics found in placental mammals [6].

With respect to extreme environmental adaptions, tenrecs tolerate exposure to hypoxic (4% O_2_) and hypercapnic (10% CO_2_) conditions well at low and high temperatures but will constrain metabolism at higher temperatures [1], possibly related to social hibernation, with as many as 13 tenrecs in a single burrow that is ~1 m deep and sealed to the surface for the ~8-month hibernation season [1,2]. In humans, however, hypoxia and hypercapnia exposure, as well as modest fluctuations in body temperature have been linked to inflammatory responses [7]. Such differences may suggest that tenrecs are likely to experience and tolerate physiological insults that would elicit a strong inflammatory cascade in humans.

Although inflammation is a well-conserved response critical to homeostasis and survival, excessive or dysregulated inflammatory responses are associated with numerous human pathologies, including obesity, neurodegeneration, autoimmune disease and cancer [8,9,10]. While inflammation coordinates responses to infection, damage and cellular stress [11], its broader evolutionary roles remain poorly understood. Comparative research suggests that key inflammatory components likely evolved from ancient stress-response pathways with initial roles in metabolic coordination and intercellular signalling [12]. In the case of Boreoeutherians, excessive inflammation may be detrimental, including cytokine storms involving IL-1B, IL-6 and TNF-α as, for example, associated with sepsis and the high mortality in the COVID-19 pandemic [13,14,15]. Excessive inflammation can contribute to widespread organ dysfunction and, therefore, it is of interest to identify whether inflammatory pathways may differ in a more ancestral like mammal, such as the tenrec, which endures frequent physiological and environmental stressors, and may, therefore, provide evolutionary information on ancient stress response pathways.

Protein citrullination (deimination) is a post-translational modification catalysed by peptidylarginine deiminases (PADs), converting protein bound arginine to citrulline, leading to structural and functional changes in target proteins. Citrullination is involved in various inflammatory (including sepsis), chronic, neurodegenerative and autoimmune diseases due to neo-epitope formation [16,17,18,19,20,21] and has roles in hypoxia and neuronal regeneration [22,23,24,25], but it also has various roles in physiological processes, including gene regulation and extracellular vesicle (EV) biogenesis [26,27,28]. The PAD family is composed of five isozymes in mammals, with PAD2 considered the most ancestral and phylogenetically conserved form across phylogeny. PADs have been identified throughout phylogeny from bacteria to mammals, with five tissue-specific PAD isozymes in mammals, three in chicken, one in bony and cartilaginous fish [29,30,31,32,33] and PAD/ADI (arginine deiminase) homologues in parasites, fungi and bacteria [34,35,36,37]. Citrullination/deimination may also allow for protein moonlighting, an evolutionary acquired phenomenon facilitating proteins to exhibit several physiologically relevant functions from within one polypeptide chain, which places it as a unique post-translational modification to study multifaceted functions of proteins in health and disease across phylogeny [16,29]. In Afrotheria, protein citrullination has been assessed in relation to metabolic and immunological function, as well as responses to hypoxic stress in the naked mole-rat [25,38]. PAD isozymes have been reported in various other Afrotheria, although no studies have been carried out on citrullinated proteins in those species, nor in relation to hibernation. In tenrecs, no studies have been published to date on protein citrullination or possible roles given tenrecs’ extreme physiology.

Extracellular vesicles (EV) are 30–1000 nm lipid bilayer structures, which are released from and taken up by cells as a critical part of cellular communication in physiological and pathobiological processes, including via transport of protein and non-coding RNA cargoes [39,40]. EV based cell communication is a mechanism conserved across phylogeny, including in bacteria, fungi, parasites and in crosstalk in host–pathogen interactions [35,37,41,42]. EVs can be isolated from most body fluids, including from plasma, and can, therefore, be useful biomarkers [39]. While EV research has mainly focussed on human pathologies and animal models of specific human diseases, there has been a recent rise in investigations of EVs’ roles in animal models across phylogeny, including species with unusual immune and metabolic features, resistance to hypoxia, ageing and cancer [38,43,44,45]. Roles of EVs in mucosal immunity during hibernation have furthermore been reported in turtles [46]. Currently, studies of animals in extreme environments are scarce and EVs have not been investigated in tenrecs to date.

microRNAs (miRs/miRNAs) are highly conserved small non-coding RNA molecules with key roles in shaping cellular communication [47]. miRs can, for example, inhibit post-transcriptional translation of mRNA as well as enhance mRNA degradation; they control gene expression and regulate biological processes [48]. miRs have been investigated in different tissues of various animal models of hibernation, including reptiles [49,50], frogs [51], bats [52], ground squirrels [53] and black bears [54]. Importantly, miRs form part of EV cargoes, and seem enriched in plasma-EVs, compared to total plasma. miR-21 is a key pro-oncogenic marker, but also with roles in fibrosis, development and metabolism [55,56,57]. miR-155 is considered a master regulator of inflammation [58], miR-210 is critical in the hypoxia response and related pathologies [59], and miR-206 has roles in metabolism and muscle growth, wound healing, and is also associated with numerous malignant diseases [60,61,62]. As research on miRs in tenrecs is scarce [63], roles in EV mediated miR transport may be of considerable interest, comparing active and hibernating states.

We hypothesise that due to the metabolic flexibility of the tenrec, this may be a useful experimental model to identify factors linked to unusual immune, stress and metabolic responses, relating to protein citrullination and EV biomarkers. The current study assessed differences in base-line citrullinome protein signatures in tenrec plasma and modifications in EV profiles, including with respect to selected microRNAs relating to inflammatory/stress, metabolic and hypoxia pathways, comparing tenrecs kept at low (12 °C) and high (28 °C) ambient temperatures, with respect to active and hibernating states.

## 2. Materials and Methods

### 2.1. Animals and Plasma Samples

Common tenrecs (*Tenrec ecaudatus*) were maintained at the University of Nevada, Las Vegas, USA (UNLV). All procedures and protocols for the animal experiments, euthanasia, sample collection and processing, were approved by the UNLV Institutional Animal Care and Use Committee. The colony originated from 40 wild-caught individuals imported from Mauritius in June 2014 under appropriate federal and state permits. Tenrecs were fed Mazuri insectivore diet (Mazuri, Saint Paul, MN, USA), supplemented with dry dog food, and provided water ad libitum.

For this study, experimental groups of hibernating versus active tenrecs, at two ambient temperatures (Ta; low versus high) were used to compare if base-line profiles of plasma citrullinomes and EV signatures (including selected EV microRNA cargoes) would differ in tenrecs which had been kept at the following four conditions: (i) hibernating tenrecs at Ta 12 °C; (ii) active tenrecs at Ta 12 °C; (iii) hibernating tenrecs at Ta 28 °C; and (iv) active tenrecs at Ta 28 °C. For sampling, animals were individually housed in cages within an environmental chamber set to ambient temperatures (Ta) of either 12 °C or 28 °C. All tenrecs were gradually acclimated from room temperature at a rate of 1–2 °C per day and maintained at their target experimental temperature for at least 3 days prior to sampling. Body temperature (Tb) was monitored using pre-calibrated iButton temperature loggers (DS1922L or DS1925L; Maxim Integrated, San Jose, CA, USA) placed against the chest with custom-designed harnesses [2]. This temperature approximates core Tb with high reliability. The recorded chest temperature is 0.47 ± 0.18 °C cooler than liver temperature across Tbs from 12.5 to 30.5 °C, N = 21, R > 0.99.

Tenrecs were euthanized via cervical dislocation followed by pneumothorax and immediately dissected on ice. Liver temperature was measured using a thermocouple to confirm Tb and is reported here. Four groups were sampled: (i) Hibernating tenrecs were sampled while torpid at Ta = 12 °C; Tb = 12.60 ± 0.06 °C, n = 3 tenrecs. (ii) Active tenrecs were sampled while active (fully mobile, eating, etc.) at Ta = 12 °C; Tb = 14.40 ± 0.49 °C, n = 3 tenrecs. (iii) Hibernating tenrecs were sampled while torpid at Ta = 28 °C; Tb = 28.06 ± 0.23 °C, n = 3 tenrecs. (iv) Active tenrecs were sampled while active at Ta = 28 °C; Tb = 32.26 ± 0.55 °C, n = 3 tenrecs. Criteria when sampling torpid tenrecs was performed as described in Treat et al., 2018 [2], during the hibernation season in June. Hibernating tenrecs have an obvious phenotype of lethargy, withdrawn eyes, prolonged anorexia, and ataxia if robustly disturbed. Studies examining Tb and metabolism confirm these animals are torpid, e.g., Tb is reduced to within ~0.5 °C of ambient temperature and is stable and metabolism is reduced as compared to active season animals [2]. It should be noted that an active season animal with a very low Tb (e.g., ~13 °C) is still fully ambulatory and alert.

**Figure 1:**
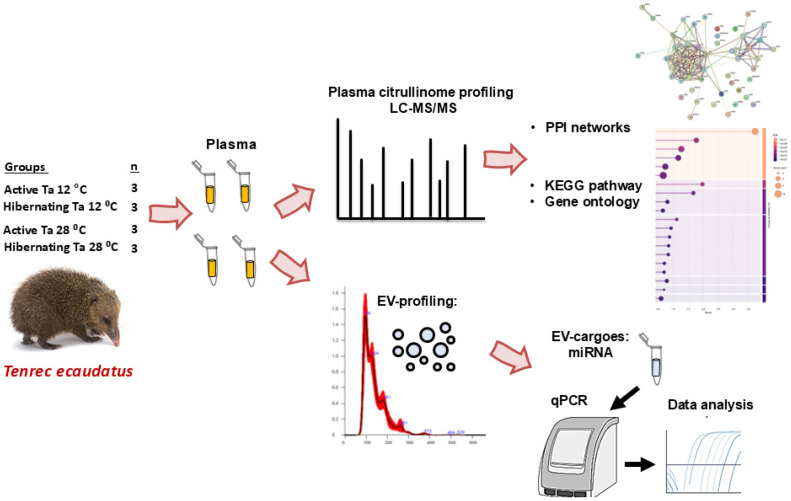
Experimental setup and workflow. Tenrecs (*Tenrec ecaudatus*) were kept at Ta 12 °C and 28 °C (n = 3 per group), and verified to be active or hibernating, respectively. Blood samples were collected and plasma isolated. Plasma citrulliome profiling was carried out by LC-MS/MS, followed by protein–protein interaction (PPI) network and functional pathway (KEGG and Gene ontology) enrichment analyses, comparing the four groups. Plasma EVs were profiled and selected miRNAs assessed in EV cargoes, comparing the four groups.

Whole blood was collected via cardiac puncture into EDTA-coated tubes, and plasma was isolated, snap frozen in liquid nitrogen, and stored at −80 °C until further analysis by Western blotting, LC-MS/MS, EV profiling and microRNA analysis. Experimental setup and workflow are shown in Figure 1.

### 2.2. Analysis of the Tenrec Plasma Citrullinomes

Citrullinated (deiminated) proteins were isolated from tenrec plasma of n = 3 samples per group (pooling 30 µL per individual sample per group) to assess representative citrullinome signatures in the hibernating versus active groups at the high and low ambient temperature conditions, respectively. The pan-citrulline F95 antibody (MABN328, Merck, Feltham, UK) was used in conjunction with the Catch-and-Release immunoprecipitation kit (17-500M, Merck) according to previously described methods [38,43]. Following overnight incubation of the plasma pools with the pan-citrulline antibody at 4 °C on a rotating platform, immunoprecipitated citrullinated proteins were eluted from the Sepharose columns using the elution buffer provided with the kit and citrullinated protein profiles assessed by SDS-PAGE and silver staining using the BioRad SilverStain kit (BioRad, Watford, UK). Citrullinated protein hits were identified by LC-MS/MS analysis following in-gel digestion (Cambridge Proteomics, Cambridge, UK).

### 2.3. LC-MS/MS and STRING Pathway Analysis of the Tenrec Plasma Citrullinomes

The citrullinated proteins isolated from the plasma of the four groups, respectively, were analysed by LC-MS/MS following in gel digestion by Cambridge Proteomics (University of Cambridge, Cambridge, UK), according to previously described methods [38]. Citrullinated protein hits were analysed both against a species-specific database of tenrec CCP_Tenrecidae Tenrecidae_20250411 (804 sequences; 243,716 residues) and against the Afrotheria database CCP_Afrotheria Afrotheria_20250414 (111,875 sequences; 64,001,564 residues), using the Mascot search algorithm (Matrix Science, London, UK). Ions score for hit identification was set at −10*Log(P), where P is the probability that the observed match is a random event, with individual ions scores > 16 for the tenrec specific database and at >37 for the Afrotheria database, to indicate identity or extensive homology (*p* < 0.05). The fragment and peptide mass tolerances were set to 0.1 Da and 20 ppm, respectively. Protein–protein interaction networks were generated by feeding protein hits for each group into the STRING database (https://string-db.org/; accessed on 15 May 2025) and analysed based on the *Homo sapiens* database. Settings were at medium confidence. Protein–protein interaction networks were compared for enrichment in pathways between the four groups for shared and distinct Gene ontology (GO), KEGG and Reactome pathways. Data for the pathway analysis of the protein networks were exported as STRING network images in png format or as Excel files.

### 2.4. Western Blotting for PAD and Histone H3 Citrullination in Tenrec Plasma and EV Surface Markers

Plasma samples were assessed (n = 3 per group) for PAD protein detection using the anti-human PAD2 isozyme-specific antibody (ab50257, Abcam, Cambridge, UK) as PAD2 is considered the most phylogenetically conserved PAD isozyme. In addition, citrullinated histone H3 was assessed in the same plasma samples using the anti-histone H3 (citrulline R2 + R8 + R17) antibody commonly used for assessment of NETosis. For the detection of plasma-EV surface markers, CD63 (ab216130) and flotillin-1 (ab41927) antibodies were used. Samples were reconstituted 1:1 in 2 × Laemmli sample buffer and boiled at 100 °C for 5 min before separation by SDS-PAGE, using 4–20% TGX gels (BioRad, Watford, UK). Proteins were blotted onto 0.45 μm nitrocellulose membranes (BioRad) using semi-dry transfer for 1 h at 15 V, with even protein transfer assessed with Ponceau S (Sigma, Gillingham, UK) staining. Membrane blocking was performed for 1 h at RT in 5% bovine serum albumin (BSA, Sigma) in Tris-buffered saline containing Tween20 (TBS-T). Primary antibody incubation was performed overnight at 4 °C on a shaking platform, diluting all primary antibodies 1/1000 in TBS-T. Membranes were washed three times in TBS-T and incubated at room temperature for 1 h in secondary antibody (anti-rabbit IgG HRP-conjugated; BioRad) diluted 1/3000 in TBS-T. Membranes were washed five times in TBS-T and then visualised using a UVP BioDoc-ITTM System (Cambridge, UK) in conjunction with ECL (Amersham Biosciences, Buckinghamshire, UK). For assessment of quantitative changes in PAD2 and CitH3 protein levels, densitometry analysis was carried out compared with PonceauS staining, using ImageJ (ij154; https://imagej.nih.gov/ij/).

### 2.5. Isolation and Characterisation of Extracellular Vesicles from Tenrec Plasma

EVs were isolated from individual plasma samples (n = 3) for each of the experimental groups (active or hibernating at Ta 12 °C; active or hibernating at Ta 28 °C), using 100 µL plasma aliquots per sample. Differential centrifugation was performed for EV isolation, adhering to the guidelines of the International Society for Extracellular Vesicles on minimal information for studies of EVs (MISEV) [39] and previously published protocols [64]. In brief, plasma was diluted 1/5 in DPBS (Dulbecco’s Phosphate-Buffered Saline) and centrifuged at 4000× *g* for 20 min, to remove any aggregates. The supernatant was collected, omitting the sediment, and centrifuged at 100,000× *g* for 1 h at 4 °C for enrichment of total EVs. Thereafter, the EV pellets were resuspended in 400 µL DPBS and centrifuged again at 4 °C for 1 h at 100,000× *g*. The supernatants were discarded, and the final washed EV pellets were resuspended in 100 µL DPBS per sample.

For nanoparticle tracking analysis (NTA), 10 µL of resuspended EVs were diluted in 990 µL DPBS, for each sample, and applied to the NTA NS300 Nanosight (Malvern Panalytical Ltd., Malvern, UK) using a syringe pump (set at 50), and five 60 s videos were recorded using the 488 nm blue laser, with capture set at 13 and post-processing set at 5. Using the NS300 software (version 3.0), replicate histograms were generated from the videos, size profiling analysis and total EV count were performed. Per individual sample, EV subgroups based on size profiling were also assessed for small EVs (≤100 nm), medium EVs (101–200 nm) and large EVs (>200 nm).

EVs were further characterised by Western blotting for two key surface markers, CD63 and flotillin-1 (as described in Section 2.4), and also imaged by transmission electron microscopy (TEM) as follows: Isolated EV pellets were resuspended in 100 mM sodium cacodylate buffer (pH 7.4), applying 3–5 μL of the EV suspension to a glow-discharged TEM grid with a carbon support film. The samples were partially air-dried for approximately 10 min before the grid was placed onto a drop of fixative solution (2.5% glutaraldehyde (Agar Scientific Ltd., Stansted, UK) in 100 mM sodium cacodylate buffer, pH 7.4) for 1 min at room temperature. The grid was subsequently transferred across three drops of distilled water for washing, with excess water being removed using filter paper. Then the grid was placed onto a drop of staining solution of 2% aqueous Uranyl Acetate (Agar Scientific Ltd., Stansted, UK) for 1 min, and any excess stain was removed with filter paper before air drying. Transmission electron microscopy (TEM) imaging of the EVs was conducted using a JEOL JEM 1400 microscope (JEOL, Tokyo, Japan) operated at 80 kV, with magnifications ranging from 30,000× to 60,000×. Digital images were captured using an AMT XR60 CCD camera (Deben, Bury Saint Edmunds, UK).

### 2.6. Analysis of miRNAs miR-21, miR-155, miR-210 and miR-206 in Tenrec Plasma-EVs

Tenrec plasma-EV signatures were assessed for four specific miRs relating to inflammation, stress and metabolism/muscle in all four sample groups. This included the pro-oncogenic miR-21, the inflammation associated miR-155, the hypoxia related miR-210 and the metabolic and muscle related miR-206. Three EV isolations were assessed per group and changes in miRNA expressions were assessed in technical triplicates. RNA was extracted from the EVs using Trizol (Sigma-Aldrich, Gillingham, Dorset, UK) and RNA purity and concentrations were assessed by NanoDrop Spectrophotometry (260 and 280 nm abs). The RNA was reverse transcribed to cDNA using the miRCURY LNA kit (Qiagen, Manchester, UK) and miRCURY LNA SYBR Green PCR Kit (Qiagen), used together with MystiCq miRNA qPCR primers for miR-21 (hsa-miR-21-5p MIRAP00047), miR-155 (hsa-miR-155-5p), miR-210 (hsa-miR-210-5p) and miR-206 (hsa-miR-206-5p). MystiCq miR-16-2-5p (MIRAP00029) and RNU6 (F 5′-GCTTCGGCAGCACATATACTAAAAT-3; R 5′-CGCTTCACGAATTT-GCGTGTCAT-3′) were used as reference genes for the normalisation of miR expression levels. miR primers were obtained from Sigma-Aldrich (Gillingham, Dorset, UK) and verified to be conserved and annotated on miRBase (http://www.mirbase.org; accessed 25 May 2025). Thermocycling conditions were as follows: denaturation at 95 °C for 2 min, 40 cycles of 95 °C for 2 s, 60 °C for 15 s, and extension at 72 °C for 15 s. For normalisation and relative expression level calculation, the 2^−ΔΔCT^ method was used according to Livak and Schmittgen [65].

### 2.7. Statistical Analysis

Each experimental group contained three plasma or plasma-EV samples for analysis. The NTA software (version 3.0, Malvern Panalytical Ltd.) was used to generate the NTA curves, showing replicates of five reads per sample, with the red line showing the standard error/deviation. STRING protein interaction networks were generated with a medium confidence setting. Ion scores in LC-MS/MS analysis were cut off for extensive homology considered at *p* < 0.05. Graph Pad Prism (version 10) was used to generate graphs comparing EV numbers and miRNA expressions, as well as densitometry readings from Western blotting analysis. The data was verified to be normally distributed using the Shapiro–Wilk test for small sample sizes and analysed using one-way ANOVA. Graphs are shown with mean and error bars representing standard deviation (SD); statistical significance was considered at *p* ≤ 0.05.

## 3. Results

### 3.1. Tenrec Plasma Citrullinomes and Pathway Enrichment Analysis in Hibernating Versus Active States at Ta 12 °C and Ta 28 °C

**Figure 2:**
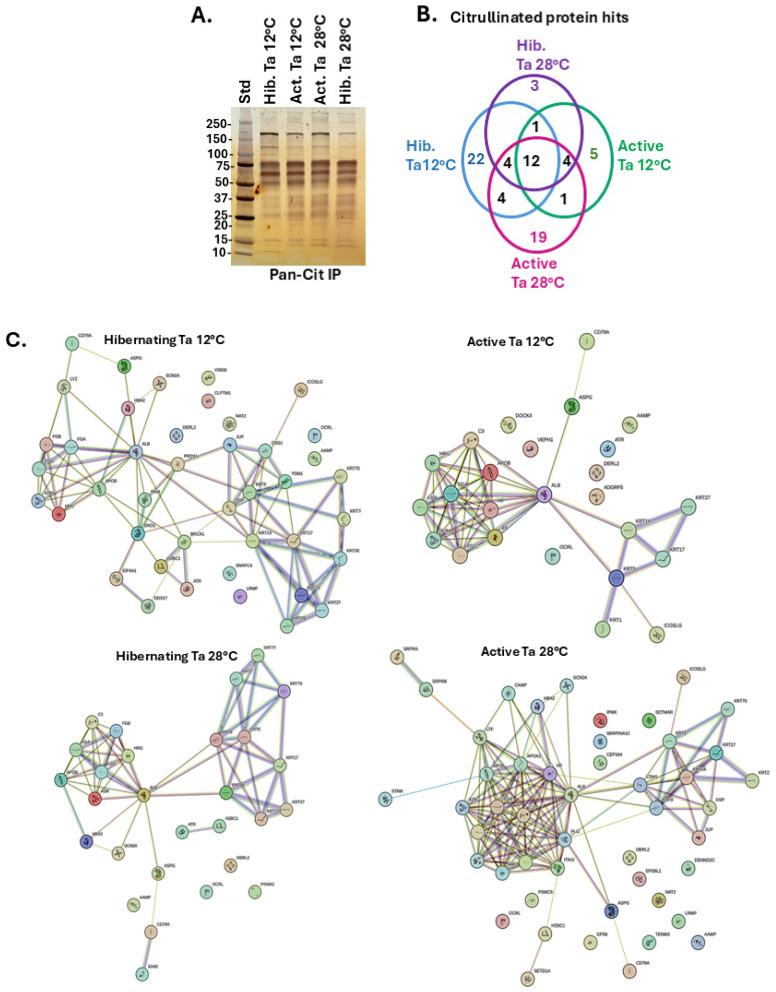

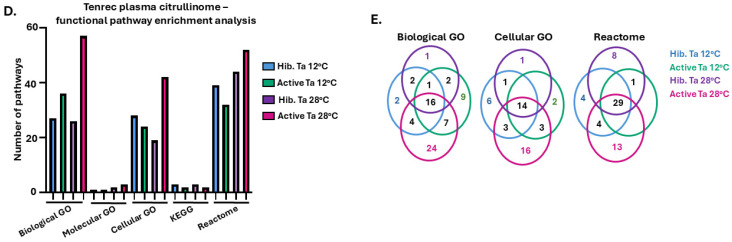
Citrullinated proteins isolated from tenrec plasma, hibernating and active conditions at Ta 12 °C and 28 °C, respectively. (**A**) A silver-stained SDS-PAGE gel, showing pan-citrulline enriched protein fractions from the four groups. (**B**) A Venn diagram summarising shared and unique citrullinated protein hits identified in the four groups by LC-MS/MS. (**C**) Protein–protein interaction (PPI) network analysis of the plasma citrullinomes of the four groups; PPI enrichment *p*-value < 1.0 × 10^−16^ for all. (**D**) Functional enrichment pathway analysis for the plasma citrullinomes of the four groups, showing Biological GO, Molecular GO, Cellular GO, KEGG and Reactome pathways. (**E**) Venn diagrams summarising numbers of shared and unique enriched pathways associated with the plasma citrullinomes of the four groups. For a full list of pathways see Supplementary Tables S2–S4.

Citrullinated proteins isolated from plasma of hibernating or active tenrecs kept at Ta 12 °C or 28 °C, respectively, were assessed by SDS-PAGE and silver staining (Figure 2A) before in-depth analysis of individual protein hits per group by LC-MS/MS analysis (for summary of protein hits see Supplementary Table S1). Differences in citrullinated protein hits between groups are presented in the Venn diagram showing shared and unique hits (Figure 2B). The associated protein–protein interaction (PPI) networks (PPI enrichment *p*-value < 1.0 *×* 10^−16^ for all) are shown for all four groups in Figure 2C. Pathway enrichment analysis associated with the PPI networks showed differences in GO, KEGG and Reactome pathways between the plasma-citrullinomes of the four groups, with overall highest numbers in pathway enrichment for the Ta 28 °C active group (Figure 2D). Shared and unique enriched pathways associated with the plasma citrullinomes of the four groups are furthermore summarised in the Venn diagrams in Figure 2E. In addition, the full lists of enriched pathways are provided in Supplementary Tables S2–S4 for Biological GO, Cellular GO, Molecular GO, KEGG and Reactome pathways, respectively.

**Figure 3:**
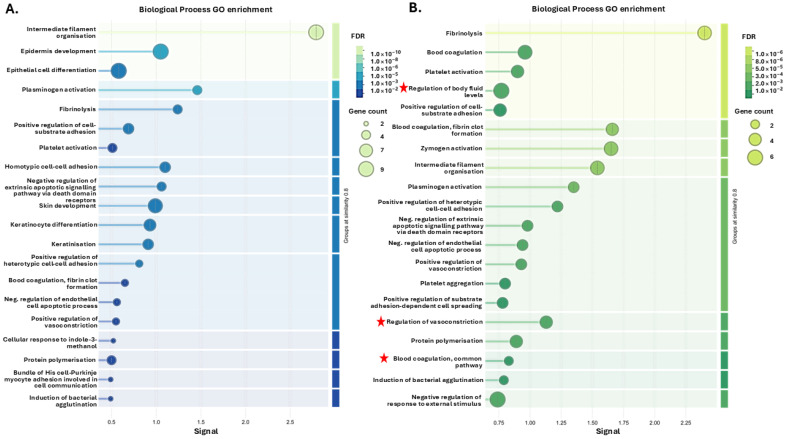

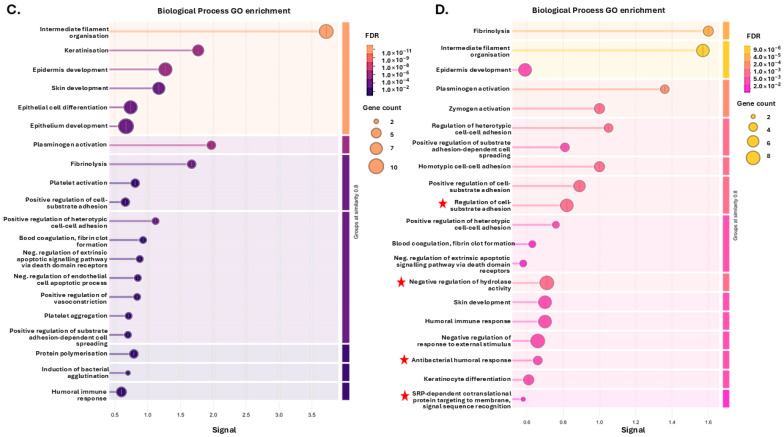
Biological process GO pathways enrichment in the plasma citrullinomes of tenrecs showing top 20 terms: (**A**) hibernating Ta 12 °C; (**B**) active Ta 12 °C; (**C**) hibernating Ta 28 °C; (**D**) active Ta 28 °C. The graphs show Signal on the *x*-axis and false discovery rate (FDR) on the *y*-axis. Gene count indicates number of proteins associated with the term. A red star indicates terms are unique to the top 20 terms identified for the respective group; see Supplementary Table S2 for full comparative analysis of all terms between the four groups.

**Figure 4:**
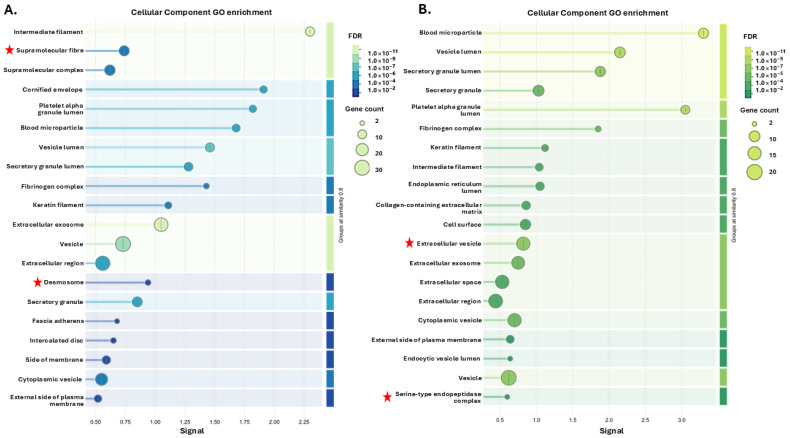

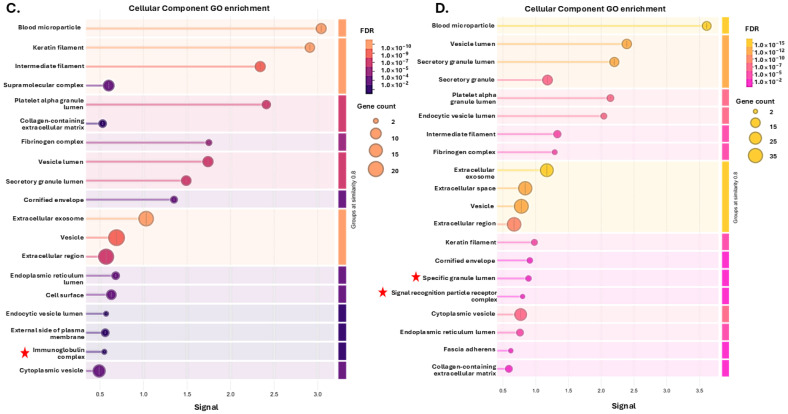
Cellular component GO pathways enrichment in the plasma citrullinomes of tenreces showing top 20 terms: (**A**) hibernating at Ta 12 °C; (**B**) active at Ta 12 °C; (**C**) hibernating at Ta 28 °C; (**D**) active at Ta 28 °C. The graphs show Signal on the *x*-axis and false discovery rate (FDR) on the *y*-axis. Gene count indicates number of proteins associated with the term. A red star indicates terms are unique to the top 20 terms identified for the respective group; see Supplementary Table S3 for full comparative analysis of all terms between the four groups.

**Figure 5:**
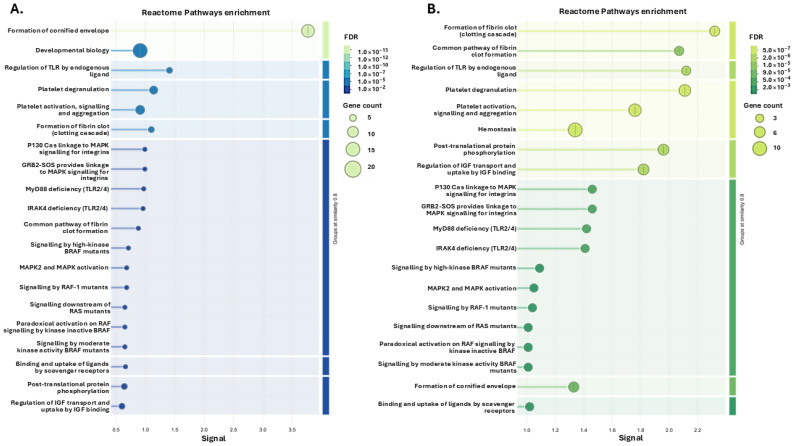

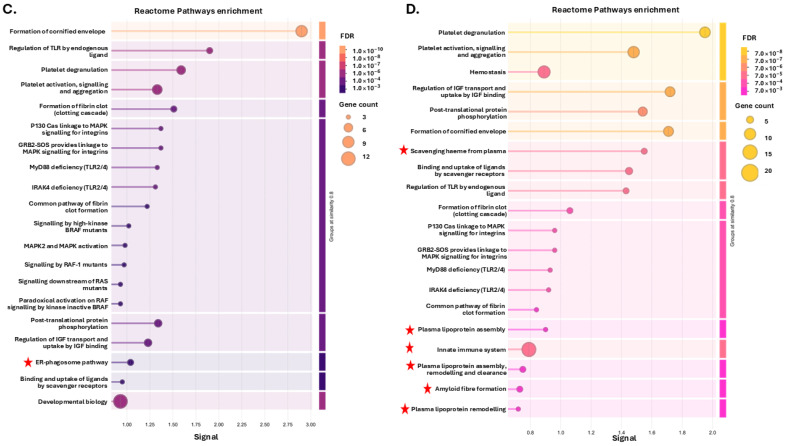
Reactome pathway enrichment in the plasma citrullinomes of tenrecs showing top 20 terms: (**A**) hibernating at Ta 12 °C; (**B**) active at Ta 12 °C; (**C**) hibernating at Ta 28 °C; (**D**) active at Ta 28 °C. The graphs show Signal on the *x*-axis and false discovery rate (FDR) on the *y*-axis. Gene count indicates number of proteins associated with the term. Differences between the 20 scoring terms are highlighted with a red star; see Supplementary Table S4 for full comparative analysis of all terms between the four groups.

For the citrullinome pathway enrichment analysis, the top 20 pathways for the GO analysis (based on signal and FDR) are presented for the four groups showing Biological process GO pathways (Figure 3), Cellular Component GO pathways (Figure 4) and Reactome GO pathways (Figure 5). A full list of all these pathways is furthermore shown for the four groups in Supplementary Tables S2–S4.

For Biological process GO pathways (Figure 3), unique pathways for the hibernating Ta 12 °C group were as follows: Developmental process and Negative regulation of apoptotic signalling pathway. For the Ta 12 °C active group unique terms were as follows: Regulation of body fluid levels, Cell activation, Blood coagulation common pathway, Blood circulation, Regulation of response to external stimulus, Regulation of response to stress, Response to other organism, Immune response and Regulation of vasoconstriction were unique GO terms associated with the plasma citrullinome. For the Ta 28 °C hibernating group, one unique Biological function GO pathway identified was Cell differentiation. The 24 unique Biological function GO pathways associated with the plasma-citrullinome of the Ta 28 °C active group included the following: Regulation of cell–substrate adhesion, Negative regulation of hydrolase activity, SRP-dependent co-translational protein targeting to membrane, signal sequence recognition, Regulation of heterotypic cell–cell adhesion, Response to stimulus, Positive regulation of apoptotic cell clearance, Negative regulation of catalytic activity, Modulation of process of another organism, Regulation of triglyceride metabolic process, Proteolysis, Wound healing, Positive regulation of cell adhesion, Biological process involved in interspecies interaction between organisms, Regulation of cell adhesion, Regulation of cell–cell adhesion, Antibacterial humoral response, Antimicrobial humoral response, Supramolecular fibre organisation, Negative regulation of cell adhesion, Regulation of cholesterol metabolic process, Negative regulation of cell–cell adhesion, Positive regulation of cellular process, Biological process involved in interaction with symbiont, and Cellular component assembly. The top 20 pathways for Biological process GO analysis of the tenrec plasma citrullinome is presented for the four groups in Figure 3; a full list of all terms, including shared and unique ones, is provided in Supplementary Table S2.

Overall, many shared terms were identified for the four groups, including within the top 20 pathways enriched, based on signal and FDR. For Cellular component GO enrichment of the plasma citrullinome in the four groups (Figure 4), Supramolecular fibre, Desmosome, Cytosol, Cytoskeleton, Ficolin-1-rich granule, and Intracellular non-membrane-bounded organelle were unique enriched terms for the Ta 12 °C hibernating group. For the Ta 12 °C active group, citrullinome enriched Cellular GO terms were Extracellular vesicle, and Serine-type endopeptidase complex. The Ta 28 °C hibernating group has one enriched term: Immunoglobulin complex. In the Ta 28 °C active group, the 16 enriched unique Cellular GO terms were Specific granule lumen, Protein-containing complex, Tertiary granule lumen, Haptoglobin–haemoglobin complex, Early endosome, Chylomicron, Intracellular organelle lumen, Tertiary granule, Endosome, Extrinsic component of plasma membrane, Intracellular anatomical structure, Very-low-density lipoprotein particle, Membrane, Polymeric cytoskeletal fibre, Signal recognition particle receptor complex, and Endocytic vesicle. The top 20 pathways for the Cellular component GO analysis of the tenrec plasma citrullinome are presented for the four groups in Figure 4; a full list of all terms, including shared and unique ones, is provided in Supplementary Table S3.

When assessing Reactome pathway enrichment for the tenrec plasma citrullinomes in the four groups (Figure 5), the Ta 12 °C hibernating group had four unique enriched terms: Meiotic synapsis, G2/M DNA damage checkpoint, Processing of DNA double-strand break ends, and Cellular response to chemical stress. No unique terms were associated with the Ta 12 °C active group. In the Ta 28 °C hibernating group, the 8 unique terms associated with the plasma citrullinome were as follows: Adaptive Immune System, CD22 mediated BCR regulation, Diseases of signal transduction by growth factor receptors and second messengers, Transport of small molecules, Signalling by the B Cell Receptor (BCR), RAF/MAP kinase cascade, Antigen activates B Cell Receptor (BCR) leading to generation of second messengers, and G2/M Checkpoints. In the Ta 28 °C active group the 13 unique Reactome pathways were as follows: Vesicle-mediated transport, Activation of C3 and C5, Heme signalling, HDL assembly, Chylomicron assembly, Chylomicron remodelling, HDL remodelling, Dissolution of Fibrin Clot, ABC transporter disorders, Scavenging by Class A Receptors, Listeria monocytogenes entry into host cells, Metabolism of proteins, and Scavenging by Class B Receptors. The top 20 pathways for the Reactome pathway enrichment analysis of the tenrec plasma citrullinome is presented for the four groups in Figure 5; a full list of all terms, including shared and unique ones, is provided in Supplementary Table S4.

### 3.2. PAD and CitH3 Detection in Tenrec Plasma in Hibernating Versus Active States at Ta 12 °C and Ta 28 °C

**Figure 6:**
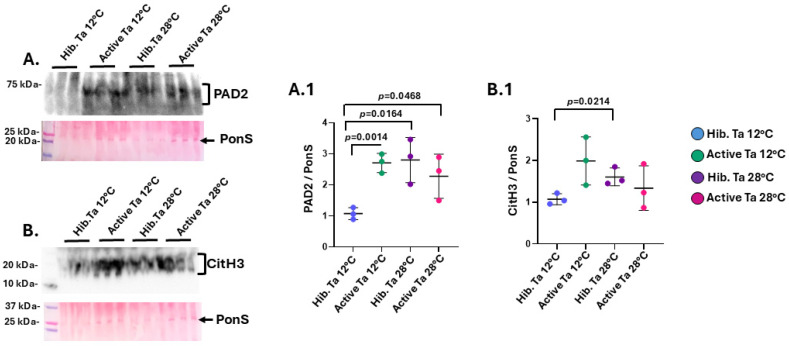
PAD2 and CitH3 protein levels in tenrec plasma comparing hibernating and active groups at Ta 12 °C and Ta 28 °C. (**A**) PAD2 levels were compared between the four experimental groups, reacting with an expected band size (indicated by the bracket) in the 70–75 kDa size range in tenrec plasma; (**A.1**) PAD2 levels were compared between the four experimental groups, densitometry analysis was based on a 25 kDa protein band (indicated by the arrow) detected by total protein staining with PonceauS. (**B**) CitH3 protein levels were compared between the four experimental groups, detecting an expected band size at approximately 20 kDa (indicated by the bracket); (**B.1**) Densitometry analysis was based on total protein detection by PonceauS (a 25 kDa band, indicated by the arrow). Exact *p*-values are shown for comparisons which reached statistical significance; One-way ANOVA, n = 3, error bars indicate SD.

For PAD protein detection in tenrec plasma by Western blotting, a band of the expected 70–75 kDa size for PAD proteins was observed by cross reaction with the human anti-PAD2 antibody (Figure 6A), which represents the phylogenetically most conserved PAD isozyme. A significant increase was observed in PAD2 levels in the active 12 °C versus the hibernating 12 °C group (Figure 6A). Furthermore, the hibernating 28 °C group showed significantly higher PAD2 levels than the hibernating 12 °C group. There were no significant differences observed in PAD2 levels between the hibernating and active 28 °C groups, but PAD2 was significantly higher in the 28 °C active than the 12 °C hibernating group (Figure 6A). When assessing changes in CitH3, indicative of NETosis as part of the inflammatory response, no significant increase in CitH3 levels was observed between active and hibernating groups, while a significant increase in CitH3 levels was observed for the 28 °C hibernating compared to the 12 °C hibernating group (Figure 6B).

### 3.3. EV Characterisation and EV Profiles in Tenrec Plasma in Hibernating Versus Active States at 12 °C and 28 °C

**Figure 7:**
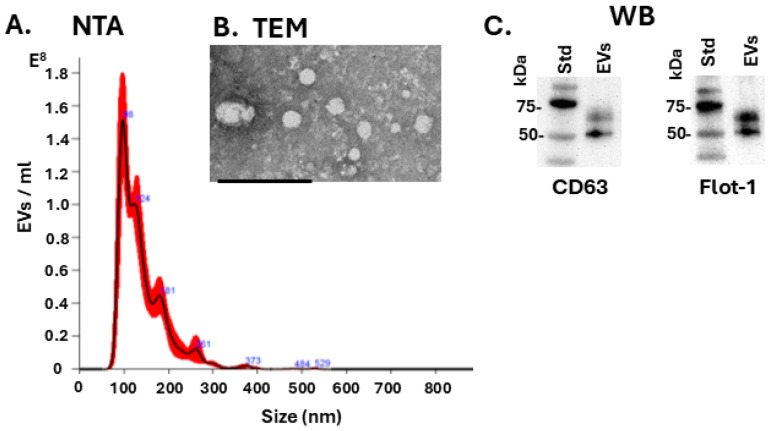
Tenrec plasma extracellular vesicle (EV) characterisation. (**A**) Nanoparticle tracking analysis (NTA) profiling, showing a representative histogram for EV size distribution and concentration; (**B**) transmission electron micrographs (TEM) showing plasma EVs (scale-bar = 100 nm); (**C**) EV surface marker detection by Western blotting (WB) for CD63 and flotillin-1, the molecular weight size standard is indicated in kDa.

Extracellular vesicles (EVs) were isolated from tenrec plasma by ultracentrifugation and characterised by nanoparticle tracking analysis (NTA), showing a size profile of approximately 50 nm to 400 nm with peaks around 100 nm; a representative NTA curve is shown in Figure 7A. The plasma-EVs were further imaged by TEM, with representative images shown in Figure 7B. The tenrec plasma-EVs were verified to be positive for the EV surface markers CD63 and Flotillin-1, as detected by Western blotting (Figure 7C).

**Figure 8:**
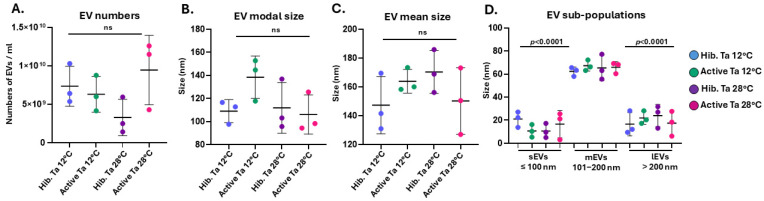
Extracellular vesicle profiling of tenrec plasma-EVs from hibernating and active animals kept at Ta 12 °C and Ta 28 °C, respectively. (**A**) Total plasma-EV numbers; (**B**) Plasma-EV modal size; (**C**) Plasma-EV mean size; (**D**) Numbers for plasma-EV subpopulations, assessing small EVs (≤100 nm), medium EVs (101–200 nm) and large EVs (>200 nm). Statistical significance was determined at *p* < 0.05 and exact *p*-values are shown (ns = non-significant); n = 3 per group, one-way ANOVA, error bars represent SD.

The plasma EV profiles were compared between the four experimental groups. Assessing total EV numbers did not reveal any significant differences between the four groups (Figure 8A). EV modal size (Figure 8B) and EV mean size (Figure 8C) showed no significant changes between groups. When assessing EV sub-populations for small EVs (≤100 nm), medium EVs (101–200 nm) and large EVs (>200 nm) the EV size distribution showed a significant increase for medium EVs in all four sample groups, confirming that this population forms the majority of tenrec plasma EVs (Figure 8D). There were no significant differences observed between the four conditions within each EV subgroup (Figure 8D).

### 3.4. Oncogenic, Inflammatory-, Hypoxia- and Metabolic-Related microRNA EV Cargoes in Tenrec Plasma of Hibernating Versus Active States at Ta 12 °C and Ta 28 °C

**Figure 9:**
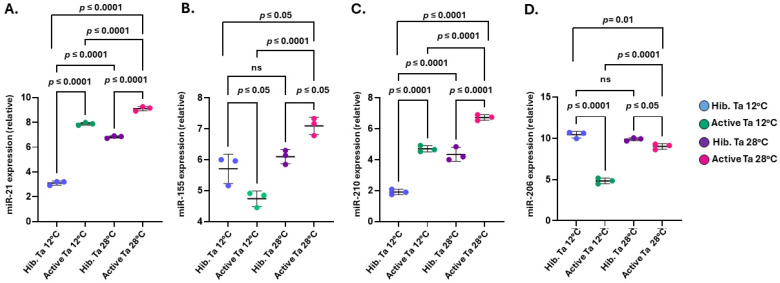
Assessment of plasma-EV microRNA cargoes in tenrecs under hibernating and active conditions at Ta 12 °C and Ta 28 °C, respectively. (**A**) Onco-related miR-21; (**B**) Inflammation-related miR-155; (**C**) Hypoxia-related miR-210; (**D**) Growth/metabolic related miR-206. Mean values with error bars representing standard deviation are presented. Relative expression compared with miR-16-2-5p expression is presented (n = 3, for each group; one-way ANOVA with error bars represent SD). Statistical significance was determined at *p* < 0.05 and exact *p*-values are shown; ns = non-significant).

The tenrec plasma-EV cargoes were assessed for four key miRs that are associated with cancer (miR-21), inflammation (miR-155), hypoxia (miR-210), metabolism and muscle function (miR-206) (Figure 9). The oncogenic miR-21 was detected at significantly higher levels in the active compared to the hibernating tenrec plasma-EVs at both temperatures tested (Ta 12 °C and 28 °C) (Figure 9A). Furthermore, comparing hibernating groups, miR-21 was significantly higher at Ta 28 °C, compared to the Ta 12 °C hibernating group. When comparing the two active groups, miR-21 was observed at significantly higher levels in the Ta 28 °C group, compared to the active Ta 12 °C group (Figure 9A; *p* ≤ 0.0001 for all). When assessing the inflammatory-related miR-155, between the two active groups, significantly higher levels were observed in the Ta 28 °C active, versus the Ta 12 °C active group (*p* ≤ 0.0001) (Figure 9B), while no significant difference was observed between the two hibernating groups. While miR-155 was significantly elevated in the active Ta 28 °C group compared to the hibernating Ta 28 °C group (*p* ≤ 0.05); the opposite was observed for the Ta 12 °C group, with miR-155 significantly lower in EVs of the active versus the hibernating animals (*p* ≤ 0.05) (Figure 9B). The hypoxia-related miR-210 was significantly higher in the active groups at both Ta 12 °C and Ta 28 °C, compared to the corresponding hibernating groups (*p* ≤ 0.0001) (Figure 9C). Comparing the hibernating groups, miR-210 was significantly higher in the Ta 28 °C hibernating group, compared with the Ta 12 °C hibernating group (*p* ≤ 0.0001) (Figure 9D). In the active groups, significantly higher levels of miR-210 were observed in the Ta 28 °C, versus the Ta 12 °C group (*p* ≤ 0.0001) (Figure 9C). When assessing the metabolic/muscle related miR-206, levels were significantly higher in plasma-EVs of the hibernating, compared with the active groups, at both Ta 12 °C (*p* ≤ 0.0001) and Ta 28 °C (*p* ≤ 0.05) (Figure 9D). No significant difference was observed between the two hibernating groups, while significantly higher miR-206 levels were observed in the active group at Ta 28 °C, compared with the active Ta 12 °C group (*p* ≤ 0.0001) (Figure 9D).

## 4. Discussion

This is the first study to assess both citrullinome and extracellular vesicle (EV) related signatures in tenrecs. With respect to protein citrullination, our findings indicate that various key inflammatory and cell signalling pathways may be regulated by this post-translational modification in tenrecs, to a varying extent at both hibernating and active states and in response to different environmental temperatures. Assessment of key inflammatory and metabolic microRNAs in EVs showed modified profiles in active versus hibernating animals, also with respect to low and high ambient temperatures.

### 4.1. The Tenrec Plasma Citrullinome Reflects Temperature and State Specific Immune Modulation

We verified that PADs, which cause protein citrullination/deimination, are detected in tenrec plasma by Western blotting, using the anti-human PAD2 antibody, reflecting the most phylogenetically conserved PAD isoform. PAD isozymes have been described in various other Afrotheria including Cape golden mole (*Chrysochloris asiatica*), African savanna elephant (*Loxodonta Africana*), Indian elephant (*Elephas maximus indicus*), small Madagascar hedgehog (*Echinops telfairi*), Cape elephant shrew (*Elephantulus edwardii*), Florida manatee (*Trichechus manatus latirostris*) and aardvark (*Orycteropus afer afer*), although no studies have been carried out on deiminated proteins in those species to date. Our findings correlate with a recent study on the naked mole-rat (*Heterocephalus glaber*), also an Afrotheria, where the presence of PAD protein and protein deimination/citrullination was verified and associated with various inflammatory and metabolic pathways [25,38]. When assessing changes in histone H3 citrullination (CitH3), indicative of NETosis as part of the inflammatory response [66], a significant increase in CitH3 levels was observed only for the Ta 28 °C hibernating tenrec group compared to the Ta 12 °C hibernating group. Our findings indicate that there is negligible inflammatory effect via NETosis comparing active versus hibernating states; while there may be increased inflammatory responses, possibly reflective of neutrophil activation and induced stress and innate immune responses comparing hibernation at higher temperatures to hibernation at lower temperatures. Interestingly, turtles have been shown to maintain innate immune responses in hibernating states [46], while attenuated NETosis was identified as a putative part of thromboprotective mechanisms in brown bears during hibernation [67].

In this context, we have previously identified that tenrecs experience profound sequestration of leukocytes to the spleen (at both active and torpid states) with a resultant leukocytopenia [68]. The availability of leukocytes in blood may range from ~0 to 77,000 white blood cells per µL while most mammals have 4500 to 11,000 white blood cells per µL. The tenrec leukocyte profile is lymphocyte-dominated, with relatively few neutrophils (6960 ± 10,101 lymphocytes per µL; 1598 ± 3451 neutrophils per µL; assessed in n = 20 tenrecs, mean ± SD tenrecs [68]). Interestingly, this profile is reminiscent of reptilian immune systems which share a lymphocyte-dominated hemogram, slower inflammatory responses which are macrophage-driven, and less destructive responses [69,70]. Since NETosis is a neutrophil-dependent event linked to PADs [71,72], the reduced availability of neutrophils in tenrecs could be associated with the modest response we observe between temperatures and states. The PAD/CitH3 attenuation in tenrecs may reflect an immune state where surveillance is intact, but inflammatory escalation is minimised.

Analysis of the plasma citrullinome revealed changes in citrullinated protein hits between the hibernating and active groups, as well as in response to the lower and higher ambient temperatures. Pathway enrichment analysis of the plasma citrullinomes showed considerable differences between the hibernating and active groups, also with respect to the lower (Ta 12 °C) and higher (Ta 28 °C) temperatures. There were though various pathway enrichment terms shared for the citrullinomes of all four groups. This included KEGG pathways relating to the complement and coagulation cascade, as well as bacterial infection (*S. aureus*), indicative of roles for citrullination in innate immunity and infection [17,31]. Various GO and Reactome pathways were also shared for the citrullinomes of all four groups and this included Biological GO terms for fibrinolysis, plasminogen activation, positive regulation of vasoconstriction, platelet activation, platelet aggregation and induction of bacterial agglutination. This further links to roles for citrullination in neutrophil-platelet association [73]. Shared Cellular GO terms for all four groups included extracellular exosome, secretory granule lumen, cytoplasmic vesicle and intermediate filament, highlighting roles for citrullination in modulating cellular communication [26,74].

Several pathways were specific for the plasma citrullinomes of the active groups: The active animals at Ta 12 °C showed enriched citrullinome associations with stress and immune response, as well as blood circulation. The active animals at Ta 28 °C showed enrichment of citrullinome associated pathways relating to apoptotic cell clearance, humoral immune response, proteolysis, cell adhesion processes, wound healing, heme signalling, and various metabolic processes (including metabolism of proteins and HDL), as well as vesicle mediated transport, activation of C3 and C5, and remodelling, scavenging by class A receptors, listeria infection, ABC transporter disorders and fibrin clot dissolution. These highlight links of citrullination to key immune and wound healing functions [75,76] and citrullination mediated modulation metabolic processes [77], as well as relevance in fibrin modulation [78]. In addition, both active groups showed further enrichment in citrullinome pathways relating to infection, blood coagulation and exocytosis, which were not associated with the hibernating groups. This may reflect different regulation of citrullination on coagulation events [79] between active and hibernating states.

Several pathways were specific for the plasma citrullinomes of the hibernating groups: Citrullinome associated pathways specific to the hibernating groups included developmental process, cell–cell adhesion, cytoskeletal associated terms, negative regulation of apoptotic signalling pathways, meiotic synapsis, G2/M DNA damage checkpoint and cellular response to chemical stress at Ta 12 °C; but cell differentiation and immunoglobulin complex, as well as adaptive immune system, disease of signal transduction by growth factor receptors, small molecules, RAF/MAP kinase cascade, and antigen activation and signalling by B cell receptor at Ta 28 °C. The KEGG Oestrogen signalling pathway was shared for the citrullinome of both hibernating groups. These findings highlight that key cell signalling and immune-related pathways are affected by citrullination in the tenrec and this links to the complex associations of citrullination in immune regulation and autoimmune disease in humans [21,80,81,82,83,84].

In canonical study systems (Boreoeutherians—modern mammals), inflammatory responses are strong [85]. For example, NETosis is a common neutrophil-driven response that contributes to robust citrullinome profiles through PAD-mediated histone modification, with CitH3 used as a common marker [71]. Excessive inflammatory responses, due to neo-epitopes as with citrullination, or due to cytokine storms, can be both markers and mediators of inflammatory tone [86]. While such response can be protective up to a certain level, excessive innate responses can cause collateral tissue damage leading to events such as ischemia–reperfusion injury, sepsis, and autoimmunity [87]. Our findings suggest that tenrecs exhibit an attenuated inflammatory response with some differences observed between hibernating and active states. Tenrecs may preserve innate immune readiness without incurring the robust responses typically associated with tissue damage and/or organ dysfunction.

### 4.2. EV-Mediated miRNA Signalling Indicates a Flexible Immune State

Plasma-EVs were assessed between the four groups with respect to changes in numbers, modal and mean size, as well as changes in numbers of sub-populations, relating to small, medium and large EVs. EV size profiling characteristics were similar across temperatures (Ta 12 °C and 28 °C) and physiological states (active and hibernating). However, the miRNA EV cargo assessed varied. EVs can mirror inflammatory landscapes by carrying pro-inflammatory and stress-response miRNAs as markers and propagators of inflammation [88,89]. Our data suggests that while EV-mediated communication remains available at different temperatures and activity states, the EV cargo-content shifted.

The onco-related miR-21 was significantly elevated in the active versus hibernating tenrec plasma-EVs in both the Ta 12 °C and 28 °C groups. Furthermore, miR-21 was significantly increased in the Ta 28 °C hibernating group, compared to the 12 °C hibernating group. The lower miR-21 levels at hibernation correlate to previous reports on decreased miR-21 levels during torpor in bats and ground squirrels, which also are hibernating mammals [90,91]. Our findings also point to that tenrecs may be more stressed during hibernation at the higher ambient temperature. A significant elevation of miR-21 was also observed in the active Ta 28 °C group, compared to the active Ta 12 °C group. miR-21 is strongly conserved throughout evolution and while many experimentally verified targets of miR-21 are tumour suppressors, miR-21 is also linked to cardiac disease and oxidative stress [92], which may be of interest for the increase observed both in the hibernating and active groups at the higher ambient temperature.

When assessing miR-155, which is a master regulator of inflammation [58], a significant increase was observed in the Ta 28 °C active, versus the 12 °C active group, indicative of elevated stress and/or inflammatory responses at the higher temperature. miR-155 was also significantly elevated in the active Ta 28 °C group compared to the 28 °C hibernating group, indicative of higher inflammatory status in the active state. This miR has multifaceted roles in modulating immunity and inflammatory pathways also associated with tumourigenesis and hypoxia [93,94]. Roles in cytokine pathways and suppression of cell growth are also suggested, as well as in sepsis [95]. In the Ta 12 °C tenrec group, miR-155 was significantly reduced in the active versus the hibernating group, possibly indicating that this miR may be a contributing factor to the “anti-inflammatory” state of tenrec at lower temperatures, which may have some relation to their unusual physiology. Interestingly, in studies of estivation in *Xenopus laevis*, miR-155 was downregulated during medium dehydration, but upregulated in response to high dehydration [51], indicating complex roles of this miR in states of torpor.

The hypoxia-related miR-210 was significantly higher in the active, compared to the hibernating groups at both Ta 12 °C and 28 °C, reflective of high ability of tolerating hypoxia. miR-210 was also significantly increased in the Ta 28 °C hibernating group, versus the 12 °C hibernating group, possibly indicative of higher stress when hibernating at the higher temperature. Likewise, miR-210 was also significantly increased in the Ta 28 °C active versus the 12 °C active group. As tenrecs are known to be hypoxia-tolerant animals and to exhibit changes in their metabolic demand in hypoxia [1,96], miR-210 may have functional roles in metabolic control, possibly contributing to their unusual physiological responses. miR-210 has important roles in regulating mitochondrial metabolism [97] and cell glycolytic activity, as well as being linked to inflammation [98]. miR-210 has been identified as a regulator of the hypoxia pathway and reported to have pro-apoptotic functions under normoxic conditions, but anti-apoptotic effects under hypoxic conditions [99,100].

When assessing the growth/metabolic-related miR-206, this was significantly higher in plasma-EVs of the hibernating, compared to the active groups, at both Ta 12 °C and 28 °C, and may indicate preservation in function of critical muscle maintenance, including skeletal and cardiac muscle [92], as well as possible metabolic contributions in the hibernating state. This would relate to findings reported on elevated miR-206 in torpid bats, where roles for muscle mass preservation and atrophy resistance are suggested during hibernation [90]. Interestingly, a significant increase in miR-206 was observed in the active group at Ta 28 °C, compared with the active 12 °C group, which may mirror more involvement in muscle related functions. In Teleostei and Actinopterygii, miR-206 has interestingly been shown to have modified expressions in response to unfavourable environmental temperatures, with a possible trade-off in immune versus growth responses [33,101].

Collectively, our data suggests that post-translational citrullination may contribute to a range of physiological and pathobiological processes, including immune related ones, in tenrecs. Furthermore, there was a considerable modification of EV miR cargo content relating to inflammatory and stress responses, which warrants further in-depth investigations. As PADs have been found to play major roles in the regulation of EV release [26,27,28], their contributions to EV-mediated cell communication in tenrec physiology, including in response to various stressors, remain to be further investigated. The use of protein-moonlighting [102] via post-translational citrullination for a wide range of physiological and immune-relevant functions may reflect an ancient, energetically efficient immune strategy, where heterothermic mammals, like tenrecs, reduce collateral damage during periods of metabolic suppression and environmental stress. As a model species, tenrecs provide an evolutionary perspective into how immune responses may have been shaped not just for defence but also for flexibility, longevity, and regeneration. Our findings may inform human pathobiology, where in many cases overreactive immune responses lead to severe disease responses, as seen, for example, in sepsis, autoimmunity, and neurodegeneration. Citrullination is known to be a significant contributor to these processes in humans, including via neo-epitope formation and NETosis [19,21,103,104,105].

Understanding biological processes and identifying biomarkers associated with how non-model species regulate immune tone during hibernation could inform approaches for human health in deep space travel, where preserving immune function while avoiding inflammation-induced damage will be essential for survival in extreme environment conditions.

## 5. Conclusions

In summary, our study provides the first characterisation of plasma citrullinome and EV profiles in the tenrec, a hibernating species with extreme physiology. We revealed novel aspects of post-translational citrullination/deimination and its potential role in modulating physiological plasticity across hibernating and active states. Our findings furthermore indicate changes in EV-mediated signalling via miRNA cargoes related to oncogenic, inflammatory, hypoxic, and metabolic pathways, comparing active and hibernating states at different temperatures. These findings may be of translational value to human physiological responses in extreme environments, including during deep space exploration.

## Data Availability

The data is contained within the article and Supplementary Materials.

## Supplementary Materials

The following supporting information can be downloaded at: https://www.mdpi.com/article/10.3390/biology14081056/s1, Tables S1–S4: Table S1. Citrullinated protein hits identified in the experimental tenrec groups, comparing with the Afrotheria database (111,875 sequences; 64,001,564 residues). Protein name, protein ID and species are indicated. A tick (V) indicates that the citrullinated protein hit was identified in the respective group; Table S2. Biological process GO pathways of the plasma citrullinome of hibernating and active groups at 12 °C and 28 °C; Table S3. Cellular component GO, Molecular function GO and KEGG pathways of the plasma citrullinome of hibernating and active groups at 12 °C and 28 °C; Table S4. Reactome pathways of the plasma citrullinome of hibernating and active groups at 12 °C and 28 °C.
