# Supplementary material for: Immune Tuning in Extreme Environments: Protein Citrullinome and Extracellular Vesicle Signatures Comparing Hibernating Versus Active States in the Heterothermic and Heterometabolic Tenrec (Tenrec ecaudatus)
Source: Biology (Basel). 2025 Aug 15;14(8):1056. doi: 10.3390/biology14081056 (PMC12383951; doi:10.3390/biology14081056)
Supplement: Supplementary file 1 [file biology-14-01056-s001.zip › Supplementary Table S1.pdf]

Supplementary Table S1. Citrullinated protein hits identified in the experimental tenrec groups, comparing with the Afrotheria database (111875 sequences; 64001564 residues). Protein name, protein ID and species are indicated. A tick (V) indicates that the citrullinated protein hit was identified in the respective group.

**Protein name**	**Protein ID**	**Species**	**Hib 12°C**	**Act 12°C**	**Hib 28°C**	**Act 28°C**
**GROUP 1 ONLY**						
Breast and ovarian cancer susceptibility 1 BRCA1	A0A165QPM3_MICTA BRCA1	*Nesogale talazaci*	V			
Keratin, type I cuticular Ha5	A0A8B6ZYX3_ORYAF	*Orycteropus afer afer*	V			
Keratin, type I cuticular Ha1	A0A2Y9EAM7_TRIMA	*Trichechus manatus latirostris*	V			
Keratin, type I cytoskeletal 16-like	A0A8B6ZXH8_ORYAF	*Orycteropus afer afer*	V			
Keratin, type I cytoskeletal 28	A0A8B6ZXG3_ORYAF	*Orycteropus afer afer*	V			
Keratin, type I cytoskeletal 42	A0A8B6ZXJ1_ORYAF	*Orycteropus afer afer*	V			
Keratin 78	G3TX30_LOXAF	*Loxodonta africana*	V			
Actin, gamma-enteric smooth muscle isoform X1	A0A2Y9D7T4_TRIMA	*Trichechus manatus latirostris*	V			
Protein-glutamine gamma-glutamyltransferase	A0A8B6ZCU2_ORYAF	*Orycteropus afer afer*	V			
phosphopyruvate hydratase	A0A2Y9E153_TRIMA	*Trichechus manatus latirostris*	V			
Ubiquitin-ribosomal protein eS31 fusion protein	A0A2Y9D7Q5_TRIMA	*Trichechus manatus latirostris*	V			
Lysozyme	A0A077S1L2_LOXAF	*Loxodonta africana*	V			
Desmoglein-1	A0A8B7AVK9_ORYAF	*Orycteropus afer afer*	V			
RNA helicase EIF4A1	A0A2Y9DJW5_TRIMA	*Trichechus manatus latirostris*	V			
Desmocollin-1 isoform X1	A0A2Y9E4T5_TRIMA	*Trichechus manatus latirostris*	V			
Peroxiredoxin-1	A0A2Y9DC61_TRIMA	*Trichechus manatus latirostris*	V			
snRNA-activating protein complex subunit 4 SNAPC4	A0A9B0TN88_CHRAS	*Chrysochloris asiatica*	V			
Pyruvate kinase	A0A2Y9QQ09_TRIMA	*Trichechus manatus latirostris*	V			

DENN domain-containing<br>protein 2C isoform X1	A0A9B0TJC2_CHRAS	*Chrysochloris asiatica*	V			
Lipid scramblase CLPTM1	A0A2Y9E7U0_TRIMA	*Trichechus manatus latirostris*	V			
V-set and immunoglobulin domain containing 8<br>VSIG8	G3T0B0_LOXAF	*Loxodonta africana*	V			
Desmoplakin	A0A9B0WRD4_CHRAS	*Chrysochloris asiatica*	V			
**GROUP 2 ONLY**						
Keratin, type II cytoskeletal 1	A0A8B7AIB5_ORYAF	*Orycteropus afer afer*		V		
Coagulation factor V	A0A2Y9RIK4_TRIMA	*Trichechus manatus latirostris*		V		
Probable G-protein coupled receptor 116<br>GPR116	A0A9B0WMU6_CHRAS	*Chrysochloris asiatica*		V		
Dedicator of cytokinesis protein 4<br>DOCK4	A0A9B0T5K0_CHRAS	*Chrysochloris asiatica*		V		
Ventricular zone-expressed PH domain-containing protein homolog 1 isoform X1<br>VEPH1	A0A2Y9DQJ7_TRIMA	*Trichechus manatus latirostris*		V		
**GROUP 3 ONLY**						
Keratin, type II cytoskeletal 2 epidermal<br>KRT2	A0A9B0TWK7_CHRAS	*Chrysochloris asiatica*			V	
Keratin, type I cytoskeletal 13	A0A2Y9G5D3_TRIMA	*Trichechus manatus latirostris*			V	
Keratin 77	G3T5Z6_LOXAF	*Loxodonta africana*			V	
**GROUP 4 ONLY**						
Serotransferrin-like<br>LOC103193724	A0A8B6ZJ99_ORYAF	*Orycteropus afer afer*				V
Plasminogen<br>PLG	G3U701_LOXAF	*Loxodonta africana*				V
Plasminogen<br>PLG	A0A2Y9D6R6_TRIMA	*Trichechus manatus latirostris*				V
Lactotransferrin<br>LTF	G3T0S5_LOXAF	*Loxodonta africana*				V
Signal recognition particle receptor subunit beta	A0A9B0T5R3_CHRAS	*Chrysochloris asiatica*				V
cathelicidin antimicrobial peptide<br>CAMP	A0A9B0TY79_CHRAS	*Chrysochloris asiatica*				V

Signal transducing adapter molecule 1 isoform X1<br>STAM	A0A2Y9DSZ5_TRIMA	*Trichechus manatus latirostris*				V
Apolipoprotein A-I<br>APOA1	APOA1_ORYAF	*Orycteropus afer*				V
Pregnancy zone protein-like<br>LOC101349741	A0A2Y9E6J0_TRIMA	*Trichechus manatus latirostris*				V
uncharacterized protein<br>LOC111820845	A0A2Y9R4A8_TRIMA	*Trichechus manatus latirostris*				V
26S proteasome regulatory subunit 8<br>PSMC5	A0A2Y9DGQ5_TRIMA	*Trichechus manatus latirostris*				V
centrosomal protein of 164 kDa<br>CEP164	A0A8B6ZGA2_ORYAF	*Orycteropus afer afer*				V
DENN domain-containing<br>protein 2C isoform X1<br>DENND2C	A0A9B0TJC2_CHRAS	*Chrysochloris asiatica*				V
Cadherin-1<br>CDH1	A0A9B0WMJ1_CHRAS	*Chrysochloris asiatica*				V
Histone-lysine N-methyltransferase<br>SETD1A	A0A9B0WYQ1_CHRAS	*Chrysochloris asiatica*				V
Complement C4-A<br>LOC101351736	A0A2Y9EBD6_TRIMA	*Trichechus manatus latirostris*				V
Serpin family A member 12<br>SERPINA12	G3U5P2_LOXAF	*Loxodonta africana*				V
Epidermal growth factor receptor kinase substrate 8<br>EPS8	A0A2Y9E5L2_TRIMA	*Trichechus manatus latirostris*				V
Inter-alpha-trypsin inhibitor heavy chain H3<br>ITIH3	A0A2Y9D6T6_TRIMA	*Trichechus manatus latirostris*				V
Teneurin-3<br>TENM3	A0A2Y9E8Q8_TRIMA	*Trichechus manatus latirostris*				V
Desmoplakin isoform X3<br>DSP	A0A2Y9E9D6_TRIMA	*Trichechus manatus latirostris*				V
**GROUPS 1 & 4**						
Keratin, type I cytoskeletal<br>10	G3U8C3_LOXAF	*Loxodonta africana*	V			V
Junction plakoglobin<br>JUP	A0A8B6ZXA9_ORYAF	*Orycteropus afer afer*	V			V
Histone H3.1-like<br>LOC101350587	A0A2Y9E0I0_TRIMA	*Trichechus manatus latirostris*	V			V

Inositol 1,4,5-triphosphate receptor associated 2 IRAG2	A0A2Y9DYI9_TRIMA	*Trichechus manatus latirostris*	V			V
**GROUPS 2 & 4**						
Haptoglobin LOC101349327	A0A2Y9RSU5_TRIMA	*Trichechus manatus latirostris*		V		
Haptoglobin HP	A0A9B0TDA5_CHRAS	*Chrysochloris asiatica*				V
Haptoglobin HP	A0A8B6ZWZ5_ORYAF	*Orycteropus afer afer*				V
**GROUPS 3 & 4**						
Keratin, type II cytoskeletal 8 KRT8	A0A2Y9DG88_TRIMA	*Trichechus manatus latirostris*			V	V
**GROUPS 1,2,3**						
Serine/threonine-protein kinase ATR	A0A2Y9RKU6_TRIMA	*Trichechus manatus latirostris*	V	V	V	
**GROUPS 1,2,4**						
Ig-like domain-containing protein	G3U800_LOXAF	*Loxodonta africana*	V	V		
Ig-like domain-containing protein	G3U8J5_LOXAF G3TSP3_LOXAF	*Loxodonta africana*				V
Ig-like domain-containing protein	G3U250_LOXAF	*Loxodonta africana*	V			
**GROUPS 1,3,4**						
Hemoglobin subunit alpha HBA	P24291 HBA_ECHTE HBA	*Echinops telfairi*	V		V	V
Hemoglobin subunit alpha	HBA_ECHTE	*Echinops telfairi*	V			
Keratin 75	G3SWG0_LOXAF	*Loxodonta africana*	V			V
Keratin 75	G3SSA6_LOXAF	*Loxodonta africana*	V		V	
Keratin, type II cytoskeletal 75	A0A9B0TSH2_CHRAS	*Chrysochloris asiatica*	V			
Histone H2B type 1-A LOC101361893	A0A2Y9DZ76_TRIMA	*Trichechus manatus latirostris*	V		V	V
**GROUPS 2,3,4**						
Gamma fibrinogen	Q6X869_TENEC	*Tenrec ecaudatus*		V	V	V
Complement C3 C3	G3T5N8_LOXAF	*Loxodonta africana*		V		V
Complement C3-like LOC103213477	A0A8B7BD60_ORYAF	*Orycteropus afer afer*			V	
Complement C3-like LOC102834923	A0A9B0U6E7_CHRAS	*Chrysochloris asiatica*				V
Complement C3	A0A9B0WUB3_CHRAS	*Chrysochloris asiatica*				V

Complement C3	A0A8B7ASL9_ORYAF	*Orycteropus afer afer*				V
Immunoglobulin heavy constant Mu IGHM	G3UMK9_LOXAF	*Loxodonta africana*		V	V	V
**GROUPS 1-4**						
Apolipoprotein B ApoB	G5CVZ0 G5CVZ0_ECHTE	*Echinops telfairi*	V	V		
Apolipoprotein B ApoB	G3T3D1_LOXAF	*Loxodonta africana*		V	V	V
Apolipoprotein B-100 APOB	A0A9B0WJH0_CHRAS	*Chrysochloris asiatica*	V			V
Apolipoprotein B-100 APOB	A0A2Y9E5N6_TRIMA	*Trichechus manatus latirostris*				V
Keratin, type II cytoskeletal 5	A0A9B0TYC3_CHRAS	*Chrysochloris asiatica*	V	V		
Keratin, type II cytoskeletal 5 KRT5	A0A2Y9E782_TRIMA	*Trichechus manatus latirostris*			V	
Keratin, type II cytoskeletal 5	G3SL91_LOXAF	*Loxodonta africana*	V			V
Keratin, type I cytoskeletal 14	G3T6A4_LOXAF	*Loxodonta africana*	V	V	V	V
Keratin, type I cytoskeletal 14	A0A2Y9EC25_TRIMA	*Trichechus manatus latirostris*	V		V	
Fibrinogen beta chain isoform X1 FGB	A0A2Y9FXG3_TRIMA	*Trichechus manatus latirostris*	V	V	V	
Fibrinogen beta chain FGB	A0A9B0WUQ9_CHRAS	*Chrysochloris asiatica*		V	V	V
Fibrinogen gamma chain isoform X2	A0A2Y9DQ56_TRIMA	*Trichechus manatus latirostris*	V	V	V	
Fibrinogen gamma chain FGG	G3TIU1_LOXAF	*Loxodonta africana*				V
Fibrinogen alpha chain	A0A2Y9DQ38_TRIMA	*Trichechus manatus latirostris*	V	V	V	V
Fibrinogen alpha chain	A0A8B7A2M2_ORYAF	*Orycteropus afer afer*				V
Fibrinogen alpha chain FGA	A0A9B0TWJ9_CHRAS	*Chrysochloris asiatica*		V	V	V
Albumin ALB	A0A2Y9RIZ4_TRIMA	*Trichechus manatus latirostris*	V	V	V	V
Albumin ALB	A0A9B0WYD1_CHRAS	*Chrysochloris asiatica*	V	V	V	V
Albumin ALB	A0A8B7B0H8_ORYAF	*Orycteropus afer afer*				V

asparaginase	A0A8B7A4D2_ORYAF	*Orycteropus afer afer*	V	V	V	V
Histidine-rich Glycoprotein HRG	A0A8B6ZES1_ORYAF	*Orycteropus afer afer*	V	V	V	V
Histidine-rich glycoprotein	A0A2Y9RB27_TRIMA	*Trichechus manatus latirostris*	V	V	V	
Alpha-2-macroglobulin	A0A8B6ZI97_ORYAF	*Orycteropus afer afer*	V	V	V	V
Alpha-2-macroglobulin	A0A2Y9RYR0_TRIMA	*Trichechus manatus latirostris*				V
IgA	A0A1S6EEF0_TRIMA	*Trichechus manatus latirostris*	V	V	V	V
