# Supplementary material for: Immune Tuning in Extreme Environments: Protein Citrullinome and Extracellular Vesicle Signatures Comparing Hibernating Versus Active States in the Heterothermic and Heterometabolic Tenrec (Tenrec ecaudatus)
Source: Biology (Basel). 2025 Aug 15;14(8):1056. doi: 10.3390/biology14081056 (PMC12383951; doi:10.3390/biology14081056)
Supplement: Supplementary file 1 [file biology-14-01056-s001.zip › Supplementary Tables S2-S4.pdf]

Supplementary Table S2. Biological process GO pathways of the plasma citrullinome of hibernating and active groups at 12°C and 28°C. Pathway enrichment was carried out for citrullinated proteins identified in plasma each group. A tick indicates that the pathway was identified in the plasma citrullinome of the respective group.

**Biological process GO term description**	**Hib. 12°C**	**Active 12°C**	**Hib. 28°C**	**Active 28°C**
Developmental process	V			
Negative regulation of apoptotic signaling pathway	V			
Regulation of body fluid levels		V		
Cell activation		V		
Blood coagulation, common pathway		V		
Blood circulation		V		
Regulation of response to external stimulus		V		
Regulation of response to stress		V		
Response to other organism		V		
Immune response		V		
Regulation of vasoconstriction		V		
Cell differentiation			V	
Regulation of cell-substrate adhesion				V
Negative regulation of hydrolase activity				V
SRP-dependent cotranslational protein targeting to membrane, signal sequence recognition				V
Regulation of heterotypic cell-cell adhesion				V
Response to stimulus				V
Positive regulation of apoptotic cell clearance				V
Negative regulation of catalytic activity				V
Modulation of process of another organism				V
Regulation of triglyceride metabolic process				V
Proteolysis				V
Wound healing				V
Positive regulation of cell adhesion				V
Biological process involved in interspecies interaction between organisms				V
Regulation of cell adhesion				V
Regulation of cell-cell adhesion				V

Antibacterial humoral response				V
Antimicrobial humoral response				V
Supramolecular fiber organization				V
Negative regulation of cell adhesion				V
Regulation of cholesterol metabolic process				V
Negative regulation of cell-cell adhesion				V
Positive regulation of cellular process				V
Biological process involved in interaction with symbiont				V
Cellular component assembly				V
Epithelial cell differentiation	V		V	
Anatomical structure development	V		V	
Homotypic cell-cell adhesion	V			V
Keratinocyte differentiation	V			V
Cellular response to indole-3-methanol	V			V
Bundle of His cell-Purkinje myocyte adhesion involved in cell communication	V			V
Zymogen activation		V		V
Negative regulation of response to external stimulus		V		V
Immune system process		V		V
Response to bacterium		V		V
Blood coagulation		V		V
Positive regulation of exocytosis		V		V
Response to external stimulus		V		V
Cellular component organization			V	V
Intermediate filament organization	V		V	V
Skin development	V		V	V
Epithelium development	V	V	V	
Humoral immune response		V	V	V
Response to stress		V	V	V
Adaptive immune response		V	V	V
Fibrinolysis	V	V	V	V
Plasminogen activation	V	V	V	V
Positive regulation of cell-substrate adhesion	V	V	V	V

Positive regulation of substrate adhesion-dependent cell spreading	V	V	V	V
Positive regulation of heterotypic cell-cell adhesion	V	V	V	V
Epidermis development	V	V	V	V
Blood coagulation, fibrin clot formation	V	V	V	V
Keratinization	V	V	V	V
Negative regulation of extrinsic apoptotic signaling pathway via death domain receptors	V	V	V	V
Negative regulation of endothelial cell apoptotic process	V	V	V	V
Positive regulation of vasoconstriction	V	V	V	V
Multicellular organismal process	V	V	V	V
Protein polymerization	V	V	V	V
Platelet activation	V	V	V	V
Induction of bacterial agglutination	V	V	V	V
Platelet aggregation	V	V	V	V

Supplementary Table S3. Cellular component GO, Molecular function GO and KEGG pathways of the plasma citrullinome of hibernating and active groups at 12°C and 28°C. Pathway enrichment was carried out for citrullinated proteins identified in plasma each group. A tick indicates that the pathway was identified in the plasma citrullinome of the respective group.

**Cellular component GO term description**	**Hib. 12°C**	**Active 12°C**	**Hib. 28°C**	**Active 28°C**
Supramolecular fiber	V			
Desmosome	V			
Cytosol	V			
Cytoskeleton	V			
ficolin-1-rich granule	V			
Intracellular non-membrane-bounded organelle	V			
Extracellular vesicle		V		
Serine-type endopeptidase complex		V		
Immunoglobulin complex			V	
Specific granule lumen				V
Protein-containing complex				V
Tertiary granule lumen				V
Haptoglobin-hemoglobin complex				V

Early endosome				V
Chylomicron				V
Intracellular organelle lumen				V
Tertiary granule				V
Endosome				V
Extrinsic component of plasma membrane				V
Intracellular anatomical structure				V
Very-low-density lipoprotein particle				V
Membrane				V
Polymeric cytoskeletal fiber				V
Signal recognition particle receptor complex				V
Endocytic vesicle				V
Supramolecular complex	V		V	
Side of membrane	V			V
Fascia adherens	V			V
Intercalated disc	V			V
Plasma membrane		V		V
Extracellular space		V		V
Endomembrane system		V		V
Secretory granule	V	V		V
Cell periphery	V	V		V
Cytoplasm	V	V		V
Cornified envelope	V		V	V
Endocytic vesicle lumen		V	V	V
Endoplasmic reticulum lumen		V	V	V
Blood microparticle	V	V	V	V
Extracellular exosome	V	V	V	V
Vesicle lumen	V	V	V	V
Secretory granule lumen	V	V	V	V
Vesicle	V	V	V	V
Extracellular region	V	V	V	V
Platelet alpha granule lumen	V	V	V	V
Cytoplasmic vesicle	V	V	V	V

Intermediate filament	V	V	V	V
Fibrinogen complex	V	V	V	V
Keratin filament	V	V	V	V
Cell surface	V	V	V	V
Collagen-containing extracellular matrix	V	V	V	V
External side of plasma membrane	V	V	V	V
**Molecular function GO term description**	**Hib. 12°C**	**Active 12C**	**Hibernating 28C**	**Active 28C**
Structural molecule activity	V	V	V	V
Structural constituent of skin epidermis			V	
Endopeptidase inhibitor activity				V
Signalling receptor binding				V
**KEGG term description**	**Hib. 12°C**	**Active 12°C**	**Hib. 28°C**	**Active 28°C**
Staphylococcus aureus infection	V	V	V	V
Oestrogen signalling pathway	V		V	
Complement and coagulation cascade	V	V	V	V

Supplementary Table S4. Reactome pathways of the plasma citrullinome of hibernating and active groups at 12°C and 28°C. Pathway enrichment was carried out for citrullinated proteins identified in plasma each group. A tick indicates that the pathway was identified in the plasma citrullinome of the respective group.

**Reactome term description**	**Hib. 12°C**	**Active 12°C**	**Hib. 28°C**	**Active 28°C**
Meiotic synapsis	V			
G2/M DNA damage checkpoint	V			
Processing of DNA double-strand break ends	V			
Cellular response to chemical stress	V			
Adaptive Immune System			V	
CD22 mediated BCR regulation			V	
Diseases of signal transduction by growth factor receptors and second messengers			V	
Transport of small molecules			V	
Signaling by the B Cell Receptor (BCR)			V	
RAF/MAP kinase cascade			V	

Antigen activates B Cell Receptor (BCR) leading to generation of second messengers			V	
G2/M Checkpoints			V	
Vesicle-mediated transport				V
Activation of C3 and C5				V
Heme signaling				V
HDL assembly				V
Chylomicron assembly				V
Chylomicron remodeling				V
HDL remodeling				V
Dissolution of Fibrin Clot				V
ABC transporter disorders				V
Scavenging by Class A Receptors				V
Listeria monocytogenes entry into host cells				V
Metabolism of proteins				V
Scavenging by Class B Receptors				V
Cell junction organization	V			V
Amyloid fiber formation	V			V
Apoptotic cleavage of cell adhesion proteins	V			V
Neutrophil degranulation	V			V
MyD88:MAL(TIRAP) cascade initiated on plasma membrane		V	V	
Disease			V	V
Post-translational protein modification			V	V
Developmental Biology	V		V	V
Cellular responses to stress	V		V	V
Extracellular matrix organization		V	V	V
Plasma lipoprotein remodeling		V	V	V
Platelet degranulation	V	V	V	V
Regulation of Insulin-like Growth Factor (IGF) transport and uptake by Insulin-like Growth Factor Binding Proteins (IGFBPs)	V	V	V	V
Formation of the cornified envelope	V	V	V	V
Platelet activation, signaling and aggregation	V	V	V	V
Post-translational protein phosphorylation	V	V	V	V

Innate Immune System	V	V	V	V
Scavenging of heme from plasma	V	V	V	V
Binding and Uptake of Ligands by Scavenger Receptors	V	V	V	V
Hemostasis	V	V	V	V
Regulation of TLR by endogenous ligand	V	V	V	V
Formation of Fibrin Clot (Clotting Cascade)	V	V	V	V
Immune System	V	V	V	V
GRB2:SOS provides linkage to MAPK signaling for Integrins	V	V	V	V
p130Cas linkage to MAPK signaling for integrins	V	V	V	V
MyD88 deficiency (TLR2/4)	V	V	V	V
IRAK4 deficiency (TLR2/4)	V	V	V	V
Plasma lipoprotein assembly	V	V	V	V
Common Pathway of Fibrin Clot Formation	V	V	V	V
Plasma lipoprotein assembly, remodeling, and clearance	V	V	V	V
Integrin cell surface interactions	V	V	V	V
ER-Phagosome pathway	V	V	V	V
Signaling by high-kinase activity BRAF mutants	V	V	V	V
MAP2K and MAPK activation	V	V	V	V
Signaling by RAF1 mutants	V	V	V	V
Signaling by moderate kinase activity BRAF mutants	V	V	V	V
Paradoxical activation of RAF signaling by kinase inactive BRAF	V	V	V	V
Signaling downstream of RAS mutants	V	V	V	V
Type I hemidesmosome assembly	V	V	V	V
Signaling by BRAF and RAF1 fusions	V	V	V	V
